# Children’s Exposures to Pyrethroid Insecticides at Home: A Review of Data Collected in Published Exposure Measurement Studies Conducted in the United States

**DOI:** 10.3390/ijerph9082964

**Published:** 2012-08-17

**Authors:** Marsha K. Morgan

**Affiliations:** National Exposure Research Laboratory, U.S. EPA, Research Triangle Park, NC 27711, USA; Email: morgan.marsha@epa.gov; Tel.: +1-919-541-2598; Fax: +1-919-541-0905

**Keywords:** exposure, children, pyrethroids, urinary biomarker

## Abstract

Pyrethroid insecticides are frequently used to control insects in residential and agriculture settings in the United States and worldwide. As a result, children can be potentially exposed to pyrethroid residues in food and at home. This review summarizes data reported in 15 published articles from observational exposure measurement studies conducted from 1999 to present that examined children’s (5 months to 17 years of age) exposures to pyrethroids in media including floor wipes, floor dust, food, air, and/or urine collected at homes in the United States. At least seven different pyrethroids were detected in wipe, dust, solid food, and indoor air samples. Permethrin was the most frequently detected (>50%) pyrethroid in these media, followed by cypermethrin (wipes, dust, and food). 3-phenoxybenzoic acid (3-PBA), a urinary metabolite of several pyrethroids, was the most frequently (≥67%) detected pyrethroid biomarker. Results across studies indicate that these children were likely exposed to several pyrethroids, but primarily to permethrin and cypermethrin, from several sources including food, dust, and/or on surfaces at residences. Dietary ingestion followed by nondietary ingestion were the dominate exposure routes for these children, except in homes with frequent pesticide applications (dermal followed by dietary ingestion). Urinary 3-PBA concentration data confirm that the majority of the children sampled were exposed to one or more pyrethroids.

## 1. Introduction

In the early 1990’s, there was a growing public concern about children’s exposures to pesticides in the United States (U.S.) and potential health risks, which led Congress to pass the Food Quality Protection Act (FQPA) of 1996 [1]. The FQPA was a landmark act that amended two prior pesticide laws, the Federal Food Insecticide, Fungicide and Rodenticide Act (FIFRA) and the Federal Food, Drug, and Cosmetic Act (FFDCA) and greatly changed how the U.S. EPA regulates pesticides today [1]. The FQPA specifically requires the U.S. EPA to consider the aggregate exposures and cumulative risks to infants and children prior to establishing pesticide tolerances in food [1]. 

Pyrethroids are synthetic pesticides commonly used to control insect pests in agricultural and residential settings in the U.S. and worldwide. First generation pyrethroids (e.g., allethrin, imiprothrin, phenothrin, prallethrin, resmethrin, and tetramethrin) were developed by the early 1970’s and typically degrade quickly in sunlight, so they are primarily applied indoors or found in products like flea collars, aerosol sprays, and foggers [2,3,4]. In the late 1970’s, the second generation pyrethroids (e.g., bifenthrin, cyhalothrin, cyfluthrin, cypermethrin, deltamethrin, esfenvalerate, fenpropathrin, and permethrin) were designed to be more photostable and are frequently used in products to control for various types of insect pests on agricultural crops, in and around residential dwellings, and on pets [2,3,4]. In addition, these pesticide products are sometimes manufactured as mixtures of other pyrethroids and/or with synergists to increase their insecticidal activity, and the composition of these products can change over time [3].

Research has indicated that children can be exposed to pyrethroids in several different types of environmental media at home and in consumed foods and beverages [5,6,7,8,9]. Once absorbed into the body, the lipophilic pyrethroids are rapidly metabolized (half-lives < 24 h) to several polar metabolites and are primarily eliminated in urine [10,11]. 

A limited number of observational exposure measurement studies have been conducted to examine children’s potential exposures to pyrethroid insecticides by sampling various media such as dust, wipes, air, food, and/or urine in residential settings in the U.S. [5,6,7,8,9,12,13,14,15,16,17,18,19,20,21]. These individual studies have been vital in understanding children’s exposures and potential health risks to pyrethroids at home, including identifying important sources and/or routes of exposure. I am unaware, however, of any published review on children’s exposures to pyrethroids at home in the U.S. 

The objectives of this review paper are to examine published findings of observational exposure measurement studies that have assessed children’s exposures to pyrethroid insecticides at their homes in the U.S., to summarize multimedia measurements data collected across these studies, to discuss major findings among the studies, and to provide important insights on major data gaps and future research needs. 

## 2. Methods

A literature review using the PubMed database (http://www.ncbi.nlm.nih.gov/pubmed) was conducted to identify studies that examined children’s exposures to pyrethroids in environmental and biological (urine) media at their homes in the U.S. from 1999 to present. 

### 2.1. Environmental Measurements Data

**Figure 1:**
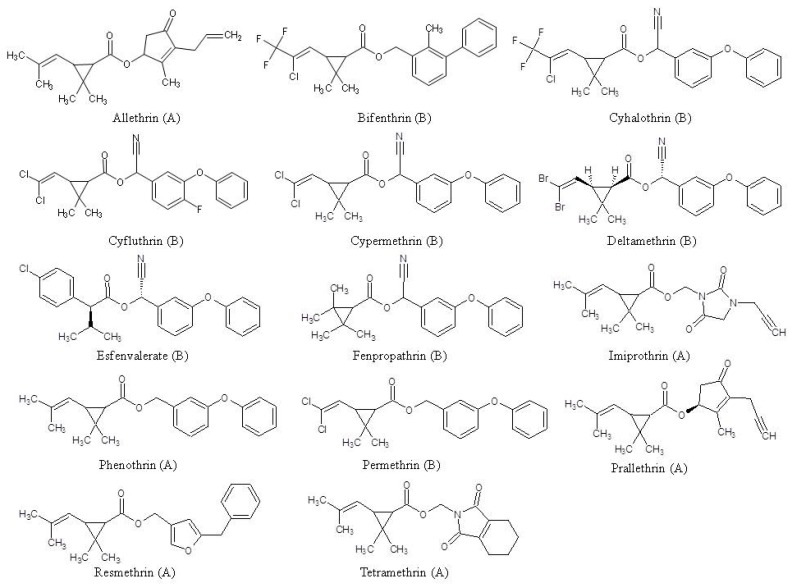
Chemical structures of the pyrethroids measured in environmental media. Parentheses denote first generation (A) or second generation (B) pyrethroid.

Table 1 presents a summary of 10 articles of studies that collected children’s environmental measurements data for pyrethroids from 2000 to 2007 [6,7,9,12,13,15,16,17,20,21]. These studies were conducted within several different states including California, Georgia, Massachusetts, North Carolina, Ohio, Washington, and Florida. Except for PETCOT [20] and CPES [9], all studies were cross-sectional having environmental measurements collected by field staff and/or participants over a 24-h or 48-h monitoring period at children’s residences. For the PEPCOT, the children’s duplicate diet samples were collected over a single 24-h monitoring period annually for three consecutive years (2003–2005). CPES collected one 24 h duplicate food sample consisting of all of the children’s consumed nonorganic fruits, fruit juices, and vegetables in two different seasons in two states (Washington and Georgia). In these studies, the children’s ages ranged from 5 months to 17 years of age, and the total number of participating children by study varied from 9 to 257. In general, the media collected, the types of methods used to collect each type of medium (*i.e.*, floor wipes, floor dust, duplicate diet, and air), and pyrethroids analyzed by medium varied among the studies. The pyrethroids that were measured in the above media in any of these studies were *cis*- and *trans*-allethrin, bifenthrin, cyfluthrin, *λ*-cyhalothrin, cypermethrin, deltamethrin, esfenvalerate, fenpropathrin, imiprothrin, *cis*- and *trans-*permethrin, phenothrin, prallethrin, resmethrin, and tetramethrin (Figure 1). The CTEPP (main study) [7] analyzed all media expect for the vacuum dust bags which were analyzed by Starr *et al.* [16]. In addition, the CTEPP (main) study data are reported in the results section by state (North Carolina (NC) and Ohio (OH)). 

### 2.2. Urinary Biomonitoring Data

Table 2 presents a summary of five articles of studies that collected children’s urinary biomonitoring measurements between 1999 and 2004 [5,7,8,17,18,19]. CPES (summer) and CPES (year) represent the same study population, however, summer season results are reported in Lu *et al*. [5] and 12 month period results, including summer season, are reported in Lu *et al.* [18]. A total of 203 children participated in the JAX-BIO [19], a biomonitoring screening study selected in an area (Jacksonville, Florida) “previously determined to have elevated rates of pesticide use”. In addition, a subset of nine of the JAX-BIO children also participated in the JAX-EXP [17], a pilot-scale observational exposure measurement study conducted at homes with known pesticide use. NHANES [8] is a nationally representative biomonitoring study while the other four studies were conducted within three states including Ohio (CTEPP), Florida (JAX-BIO and JAX-EXP), and Washington (CPES). All of these studies were cross sectional, except CPES which was a longitudinal study conducted over a 15-day period over four consecutive seasons. Spot urine voids were collected in each study, however NHANES, JAX-BIO and JAX-EXP collected one void per child, CTEPP-OH collected up to 6 urine voids for each child over a 48-h monitoring period, and CPES collected two urine voids daily (morning and bedtime) each sampling day per child. The number of children varied from 9 to 580 by study, and their ages ranged from 2–11 years of age. Only non-adjusted urine (ng/mL) data are presented since creatinine correction of urine may not be a reliable adjustment measure for children [22,23]. 

**Table 1:** Summary of observational exposure measurement studies that collected environmental samples at children’s homes in the U.S.

Reference	Study Name ^a^	Location	Year	N ^b^	Age (years)	Media Collected	Sampling Frequency	Analytical Method ^e^	Pyrethroids Measured
Bradman *et al*. [6]	CHAMACOS-QEA	Salinas Valley of Monterey California	2002	20	0–2	Outdoor air	24-h integrated sample; at 2.5 L/min	Gas chromatograph/mass spectrometer in the selected ion monitoring mode	*cis*-and *trans*-Allethrin, bifenthrin, cyfluthrin, *λ*-cyhalothrin, cypermethrin, deltamethrin, esfenvalerate, *cis*- and *trans-*permethrin, phenothrin, resmethrin, tetramethrin
						Indoor air	24-h integrated sample; at 2.5 L/min		
						Carpet dust	High volume small surface sampler (HVS3); 1 m^2^ area of carpet of main living area		
						Hard floor surface wipe	SOF-WICK pad wetted with 10 mL 2-propanol; sample collected in a 900 cm^2^ area in kitchen or dining room		
						Solid food	24-h duplicate diet sample		
						Liquid food	24-h duplicate diet sample		
						Surface Press	3M Empore disks pressed on a hard floor for 5-min		
						Clothing	Clothing worn for 3–4 h		
Chuang and Wilson [20] ^c^	PEPCOT	Raleigh-Durham-Chapel Hill area of North Carolina	2003–2005	50	3	Solid food	24-h duplicate diet sample; one sample collected annually for three consecutive years	Gas chromatograph/mass spectrometer in the selected ion monitoring mode	Bifenthrin, cyfluthrin, *λ*-cyhalothrin, cypermethrin, deltamethrin, esfenvalerate, fenpropathrin, fenvalerate, *cis*- and *trans-*permethrin, phenothrin, resmethrin, tetramethrin
				51	<3				
Julien *et al.* [15]	HPHI	Boston, Massa-chusetts	2002–2003	42	4–17	Dust (carpets & sofas combined),	9 A Eureka Mighty-mite vacuum cleaner sampled ‘all accessible surfaces’ in living rooms.	Gas chromatograph/mass spectrometer in the multiple ion monitoring mode	Bifenthrin, cyfluthrin, *λ*-cyhalothrin, cypermethrin, deltamethrin, esfenvalerate, permethrin, phenothrin, resmethrin, tetramethrin
						Hard floor surface wipes	SOF-WICK pad wetted with 5 mL of 2-propanol; sample collected each in a 929 cm^2^ area in kitchen and living room		
Lu *et al.* [9] ^d^	CPES	Seattle, Washington	2003–2004	23	3–11	Solid and liquid foods	24-h duplicate food samples consisting of nonorganic fruits, fruit juices, and vegetables. One 24-h food sample collected in the summer and fall of 2003.	Gas chromatograph with a halogen specific detector and/or a mass spectrometer	Bifenthrin, cypermethrin, esfenvalerate, permethrin
		Atlanta, Georgia	2006–2007	23	3–12		24-h duplicate food samples consisting of nonorganic fruits, fruit juices, and vegetables. One 24-h food sample collected in the summer 2005 and winter 2006.		
Morgan *et al.* [7,12] ^d^	CTEPP (main study)	North Carolina & Ohio (6 counties in each state)	2000–2001	257	2–5	Soil	Top 0.5 cm of 0.1 m^2^ area of soil where child played most often	Gas chromatograph/mass spectrometer in the selected ion monitoring mode	Cyfluthrin, *cis*- and *trans*-permethrin
						Outdoor air	48-h integrated sample; at 4.0 L/min		
						Indoor air	48-h integrated sample; at 4.0 L/min		
						Carpet dust	HVS3; 0.76 m^2^ area of carpet in most used room		
						Solid food	48-h duplicate diet sample		
						Liquid food	48-h duplicate diet sample		
						Hard floor surface wipe	SOF-WICK pad wetted with 2 mL of 2-propanol on a 0.14 m^2^ area of floor child spent most time.		
						Transferable residues	Polyurethane foam roller method; 1 roller to sample 3 different locations where child spent most time indoors		
Quandt *et al.* [13]	CFW	Western North Carolina and Virginia	2001	41	1–7	Wipes (hand, hard floor and toy)	SOF-WICK pad wetted with 10 mL of 2-propanol for 3 different wipes (all surfaces of hands, 0.42–0.84 m^2^ area of floor, mainly kitchens and hallways; samples collected from 2–3 non-plush toys	Gas chromatograph/ mass spectrometer	Esfenvalerate and *cis*- and *trans*-permethrin
Quiros-Alcala *et al.* [21] ^d^	PDLHS	Salinas and Oakland, California	2006	15	3–6	Carpet dust	HVS3; 1–2 m^2^ area of carpet where child spent time playing	Electron impact gas chromatograph/mass spectrometer in multiple ion detection mode	Allethrin, bifenthrin, cypermethrin, deltamethrin, esfenvalerate, imiprothrin, *cis*-permethrin, *trans*-permethrin, phenothrin, prallethrin
				13					
Starr *et al.* [16]	CTEPP-VAC	North Carolina & Ohio (6 counties in each state)	2000–2001	85	2–5	Vacuum dust bags	Existing bag from homeowners vacuum cleaner	Gas chromatograph/mass spectrometer in the selected ion monitoring mode	Allethrin, cyfluthrin, *λ*-cyhalothrin, cypermethrin, deltamethrin, esfenvalerate, fenpropathrin, imiprothrin, *cis*-permethrin, *trans*-permethrin, phenothrin, prallethrin, resmethrin, tetramethrin
Tulve *et al.* [17]	JAX-EXP	Jacksonville, Florida	2001	9	4–6	Outdoor air	24-h integrated sample; at 3.8 L/min	Gas chromatograph/mass spectrometer in the multiple ion monitoring mode	*cis*-and *trans*-Allethrin, bifenthrin, cyfluthrin, *λ*-cyhalothrin, cypermethrin, deltamethrin, esfenvalerate, *cis*- and *trans-*permethrin, phenothrin, tetramethrin
						Indoor air	24-h integrated sample; at 3.8 L/min		
						Hard floor wipe	SOF-WICK pad wetted with 10 mL of 2-propanol; sample collected each in a 929 cm^2^ in child’s play area and recent pesticide application area		
						Solid food	24-h duplicate diet sample		
						Clothing (socks)	Socks worn for 1 or more hours		

^a^ CHAMACOS-QEA (Center for the Health Assessment of Mothers and Children of Salinas Quantitative Exposure Assessment Study); CFW (Children of Farm workers Study); CPES (Children’s Pesticide Exposure Study in Washington and Georgia by Year), CTEPP (Children’s Total Exposure to Persistent Pesticides and Other Persistent Organic Pollutants Study, *Main Study*), CTEPP-VAC (Vacuum Cleaner Bags from the CTEPP *Main Study*); HPHI (Healthy Public Housing Initiative Study); JAX-EXP (Biological and Environmental Monitoring for Organophosphate and Pyrethroid Pesticide Exposures in Children Living in Jacksonville, Florida Study); PDLHS (Pesticides in Dust in Low-Income Households from Agricultural and Urban Communities Study); and PEPCOT (The Pesticide Exposures of Preschool Children Over Time Study). All studies are cross-sectional, except the CPES and PEPCOT. CPES was a longitudinal study conducted in the summer and fall of 2003 in Seattle, Washington and in the summer 2005 and winter 2006 in Atlanta, Georgia. PEPCOT was a longitudinal study conducted over a three year period (2003–2005) in North Carolina; ^b^ Subject-level; ^c^ Both an older and younger sibling from the same household participated in the three year longitudinal study. The older child was 3 years of age and their sibling less than 3 years of age at enrollment; ^d^ Study conducted in two different states or areas in a state; ^e^ See Supplemental Tables S1–S4 for limits of detection for pyrethroids measured in floor dust, floor wipe, solid food, and indoor air samples.

**Table 2:** Summary of observational exposure measurement studies that collected urine samples from children at their residents in the U.S.

References	Study Name ^a^	Location	Year of study	N	Age (years)	Collection Method	Frequency of Collection	Analytical Method	Measured Urinary Metabolites ^b^
Barr *et al*. [8]	NHANES	National	1999–2000	483	6–11	Spot sample	One convenience sample	High performance liquid chromatograph- electrospray chemical ionization/tandem mass spectrometer	3-PBA, 4-F-3-PBA, *cis*-DCCA, *trans*-DCCA, *cis*-DBCA
			2001–2002	580	6–11				
Lu *et al*. [5,18] ^c^	CPES (summer)	Seattle, Washington	2003	23	3–11	Spot samples	One morning void and one bedtime void each day for 15 consecutive days; each sample analyzed separately	Gas chromatograph with a halogen specific detector and/or mass spectrometer	3-PBA, 4-F-3-PBA, *cis*-DCCA, *trans*-DCCA, *cis*-DBCA
	CPES (year)	Seattle, Washington	2003–2004	23					
Morgan *et al.* [7]	CTEPP-OH	Ohio (six counties)	2001	127	2–5	Spot samples	Up to 6 spot urine samples (morning void, after lunch, and after dinner/before bedtime); samples pooled over a 48-h period	Gas chromatograph/mass spectrometer in the selected ion monitoring mode	3-PBA
Naeher *et al.* [19] ^d^	JAX-BIO	Jacksonville, Florida	2001	203	4–6	Spot sample	One convenience void	High performance liquid chromatograph-atmospheric pressure chemical ionization/ tandem mass spectrometer	3-PBA, 4-F-3-PBA, *cis*-DCCA, *trans*-DCCA, *cis*-
Tulve *et al.* [17] ^d^	JAX-EXP	Jacksonville, Florida	2001	9	4–6	Spot sample	One morning void	Gas chromatograph/mass spectrometer in the multiple ion monitoring mode	3-PBA, 4F-3-PBA, *cis*-DCCA, *trans*-DCCA

^a^ CPES (Children’s Pesticide Exposure Study in Washington in Summer or Year), CTEPP-OH (Children’s Total Exposure to Persistent Pesticides and Other Persistent Organic Pollutants Study, Ohio only), JAX-BIO (Biological Monitoring for Organophosphate and Pyrethroid Pesticide Exposures in Children Living in Jacksonville, Florida Study); JAX-EXP (Biological and Environmental Monitoring for Organophosphate and Pyrethroid Pesticide Exposures in Children Living in Jacksonville, Florida Study); and NHANES (National Health and Nutrition Examination Survey); ^b^ 3-Phenoxybenzoic acid (3-PBA); *cis*-3-(2,2-dichlorovinyl)-2,2-dimethylcyclopropane carboxylic acid (*cis*-DCCA); *trans*-3-(2,2-dichlorovinyl)-2,2-dimethylcyclopropane carboxylic acid (*trans*-DCCA); 4-fluoro-3-phenoxybenzoic acid (4F-3-PBA); *cis*-3-(2,2-dibromovinyl)-2,2-dimethylcyclopropane carboxylic acid (*cis*-DBCA); ^c^ CPES is a longitudinal biomonitoring study. CPES (summer) and CPES (year) are the same study; summer season results reported in Lu *et al.* [5] and 12 month period (including summer season) reported in Lu *et al.* [18]; ^d^ A total of 203 children participated in the Naeher *et al.* [19] study and a subset (n = 9) of them participated in the observational exposure measurements component by Tulve *et al.* [17]. This study site was selected as it was ‘previously determined to have elevated rates of pesticide use’ [19]. Tulve *et al.* [17] then recruited participants within this study who had reported pesticide use at home.

Environmental samples and urine samples were collected concurrently in the CPES, CHAMACOS-QEA, CTEPP-NC, CTEPP-OH, PEPCOT, and JAX-EXP. However, only CPES, CTEPP-OH and JAX-EXP have published data on the levels of pyrethroids in environmental media and urinary pyrethroid metabolites in children to date.

**Figure 2:**
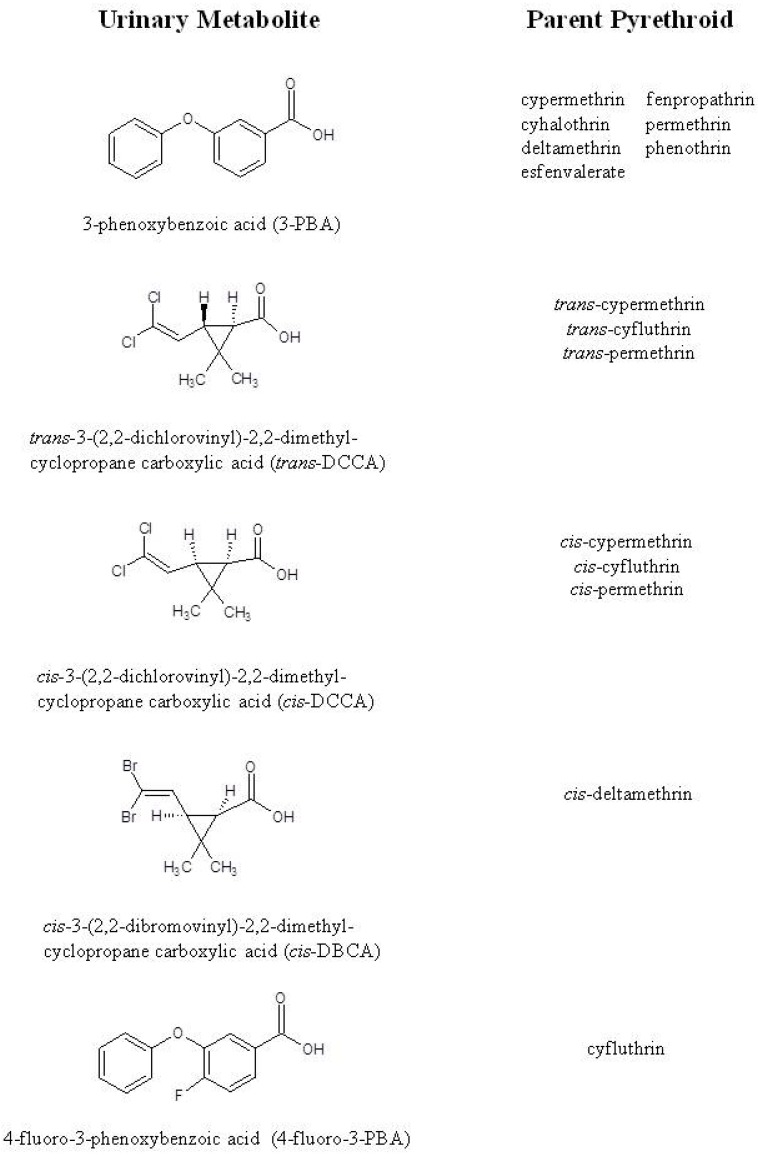
Pyrethroid metabolites and their corresponding parent pyrethroid(s) that were measured in children’s urine samples.

Up to five urinary pyrethroid metabolites were measured in children’s urine samples from the above published studies (Figure 2). These urinary metabolites include 3-phenoxybenzoic acid (3-PBA), *cis*-3-(2,2-dichlorovinyl)-2,2-dimethylcyclopropane carboxylic acid (*cis*-DCCA), *trans*-3-(2,2-dichlorovinyl)-2,2-dimethylcyclopropane carboxylic acid (*trans*-DCCA), 4-fluoro-3-phenoxybenzoic acid (4F-3-PBA), and *cis*-3-(2,2-dibromovinyl)-2,2-dimethylcyclopropane carboxylic acid (*cis*-DBCA). The urinary pyrethroid metabolites, 3-PBA, *cis*-DCCA, and *trans*-DCCA are nonspecific and confirm exposure to one or more pyrethroids [24,25]. 4-F-3-PBA and *cis*-DBCA are specific and confirm exposure to cyfluthrin and deltamethrin, respectively [25]. 

## 3. Results and Discussion

Reported here are the levels of pyrethroids measured in environmental or biological media that could be compared among three or more of the studies listed in Table 1 and Table 2. 

**Figure 3:**
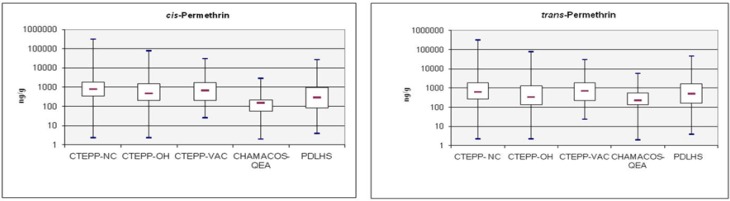
Box-and-whisker plots of the levels of *cis*- and *trans*-permethrin in floor dust samples across the studies.

### 3.1. Levels of Pyrethroids in Environmental Media

CTEPP-NC, CTEPP-OH, CTEPP-VAC, CHAMACOS-QEA, HPHI, and PDLHS used the standardized vacuum method [26] known as the high-volume small surface sampler (HVS3) to collect dust samples (ng/g) from 0.76–2 m^2^ areas of carpeted floors from various areas inside homes (living rooms, main play areas, and couches), except CTEPP-VAC and HPHI. CTEPP-VAC collected existing vacuum bags from participants, while the HPHI used a 9 A Eureka Mighty-mite vacuum cleaner to collect dust from carpets and sofas in living rooms. Surface loading measurements (ng/cm^2^) for pyrethroids in floor dust were also calculated in the CTEPP-NC, CTEPP-OH, and PDLHS. Dust concentrations (ng/g) are often used for source characterization purposes or in the estimation of ingested doses of a pesticide in health risk assessments where as surface loading measurements (ng/cm^2^) are more suitable for use in human exposure assessments [27]. Up to 12 different pyrethroids (allethrin, bifenthrin, cyfluthrin, cyhalothrin, cypermethrin, deltamethrin, esfenvalerate, imiprothrin, permethrin, phenothrin, prallethrin, and tetramethrin) were detected in floor dust samples collected at residences from several states including North Carolina, Ohio, California, and Massachusetts (Supplemental Information; Table S1). Permethrin was detected in ≥85% of the floor dust samples in all six studies. Besides permethrin only a few other pyrethroids, namely, allethrin (PDLHS), cyfluthrin (CTEPP-OH), and cypermethrin (HPHI and PDLHS), were detected above 50% in the floor dust samples. The median levels of *cis*-permethrin and *trans*-permethrin in floor dust samples varied substantially by study and ranged from 150–804 ng/g and 230–711 ng/g, respectively (Figure 3). In particular, the median levels of *cis*- and *trans*-permethrin were at least three times higher in homes (804 and 629 ng/g) in CTEPP-NC (North Carolina) than homes (150 and 230 ng/g) in CHAMACOS-QEA (California) which may reflect geographical differences in permethrin usage. For studies with reported dust surface loadings, median levels of *cis*- and *trans*-permethrin were 1,030 and 853 ng/cm^2^ (CTEPP-NC), 447 and 295 ng/cm^2^ (CTEPP-OH), and 768 and 1,620 ng/cm^2^, (PDLHS, California), respectively. These above data show that permethrin was frequently detected in floor dust samples, and that the levels of permethrin in dust (amount and loadings) at residences varied greatly by geographical region. 

The method for collecting floor wipe samples varied among the studies (CTEPP-NC, CTEPP-OH, JAX-EXP, CFW, CHAMACOS-QEA, and HPHI); however, all studies used a Johnson & Johnson SOF-WICK pad (58 to 100 cm^2^) wetted with 2 to 10 mL of 2-propanol to wipe a designated area of hard floor surface inside homes. As the collection methods were vastly different among the studies, the levels of pyrethroids measured in the wipe samples were likely influenced by such factors as the amount of 2-propanol used on the wipe, location of wipe collection, wiping method, and pesticide usage at home [28]. Up to 11 different pyrethroids (allethrin, bifenthrin, cyfluthrin, cyhalothrin, cypermethrin, deltamethrin, esfenvalerate, permethrin, phenothrin, resmethrin, and tetramethrin) were detected in floor wipes collected in the six studies from California, Florida, North Carolina, Massachusetts, Ohio, and Virginia (Table S2). Only cypermethrin (JAX-EXP and HPHI, only) and permethrin (all six studies) were detected ≥50% of the floor wipe samples. The median levels of cypermethrin in the floor wipe samples were 0.38 ng/cm^2^ in the HPHI and 0.7 ng/cm^2^ in the JAX-EXP. The median levels of *cis*-permethrin and *trans*-permethrin ranged from 0.01–0.10 ng/cm^2^ and 0.01–0.23 ng/cm^2^, respectively, among these studies. However, the median levels of *cis*- and *trans*-permethrin were about three and five times greater, respectively, in floor wipes samples from CHAMACOS-QEA than CTEPP-NC, CTEPP-OH, and JAX-EXP. CHAMACOS-QEA children lived in an agricultural area (Salinas Valley, CA, USA) which may have contributed to the higher levels of permethrin in their floor wipe samples.

CHAMACOS-QEA, CPES, CTEPP-NC, CTEPP-OH, JAX-EXP, and PEPCOT measured the levels of selected pyrethroids in duplicate diet samples that were composited over a 24-h or 48-h monitoring period. Duplicate amounts of foods and beverages consumed by each child were collected in separate containers, respectively, in all studies except CPES. CPES collected duplicate amounts of all individual fruits, vegetables, and fruit juices consumed by each child. Individual food items that weighed greater than 50 g were analyzed separately (60%), and food items less than 50 g were composited together within the same meal and then analyzed. Both an older and younger sibling from the same household participated in the PEPCOT (PEPCOT-O and PEPCOT-Y). Pyrethroids were not frequently detected (<20%) in the liquid food samples. In contrast, up to seven different pyrethroids (bifenthrin, cyfluthrin, cypermethrin, deltamethrin, permethrin, phenothrin, and tetramethrin) were detected in the children’s composited duplicate solid food samples (Table S3). Only *cis*- and *trans*-permethrin were detected in >50% of the samples from the JAX-EXP, PEPCOT-O, and PEPCOT-Y. Cypermethrin was detected in >50% of the samples only in the JAX-EXP. The median levels of *cis*- and *trans*-permethrin were fairly similar for JAX-EXP (0.29 and 0.22 ng/g), PEPCOT-O (0.15 and 0.14 ng/g) and PEPCOT-Y (0.10 and 0.09 ng/g), respectively. However, the median levels of *cis*- and *trans*-permethrin were about three times higher in children’s duplicate diet samples in JAX-EXP (0.29 and 0.22 ng/g) compared to CTEPP-NC (both <0.08 ng/g). Geographical and demographic factors (e.g., socioeconomic status and ethnicity) can influence children’s food choices and may have contributed to the higher dietary exposures of JAX-EXP children to permethrin than CTEPP children [29]. Permethrin was not detected in CHAMACOS-QEA, but this was likely due to the much higher limit of detection (4.5 ng/g) compared to the other above studies (<0.5 ng/g). For the three-year longitudinal PEPCOT study conducted in North Carolina, the median levels of *cis*- and *trans*-permethrin in solid food samples were fairly similar between the older siblings (range 0.07–0.26 and 0.07–0.37 ng/g) and younger siblings (range 0.06–0.15 and 0.06–0.16 ng/g) by year. This information suggests that PEPCOT siblings in the same household likely consumed similar types of foods and where probably exposed to similar levels of pyrethroids in their diets over a three-year period. In the CPES, 29% of the individual food items had detectable levels of pyrethroids. Pyrethroids were measureable in strawberries (bifenthrin), cherries (esfenvalerate), apples (permethrin), and broccoli (permethrin). These findings are similar to results obtained in the Food and Drug Administration’s Total Diet Study (1991–2003) which also detected measureable levels of bifenthrin, cypermethrin, esfenvalerate, and permethrin residues in certain types of foods purchased at local supermarkets and grocery stores in four different geographical regions (West, North Central, South and Northeast) of the U.S. [30]. The foods that had the highest maximum levels of residues were collards, spinach, and strawberries (bifenthrin), spinach and collards (cypermethrin), collards and apricots (esfenvalerate), and spinach and collards (permethrin). This suggests that children are likely being intermittently exposed to pyrethroids in consumed foods, particularly when eating certain types of fruits and vegetables. 

The levels of pyrethroids were measured in both indoor and outdoor air samples over a 24-h or 48-h monitoring period using various methods at residences in CTEPP-NC, CTEPP-OH, JAX-EXP, and CHAMACOS-QEA. In these studies, the frequencies of detection for up to seven pyrethroids (allethrin, bifenthrin, cyfluthrin, *λ*-cyhalothrin, cypermethrin, deltamethrin, esfenvalerate, permethrin, phenothrin, resmethrin, and tetramethrin) were <31% in the outdoor air samples in all studies except for JAX-EXP (Table S4). For JAX-EXP, *cis*- and *trans*-permethrin were both detected in 100% of the outdoor air samples with median concentrations of 2.1 and 2.5 ng/m^3^, respectively. There were also low frequencies of detection (<34%) for these pyrethroids in indoor air samples, except for permethrin. The frequencies of detection for *cis*- and *trans*-permethrin in indoor air samples varied greatly among the studies. The frequencies of detection for *cis*- and *trans*-permethrin were both higher for CTEPP-NC (>65%) than for CTEPP-OH (<23%). However, this was probably due to the higher limit of detection for permethrin in indoor air samples from CTEPP-OH (0.3 ng/m^3^) than for CTEPP-NC (0.09 ng/m^3^). The frequencies of detection in CHAMACOS-QEA were higher for *cis*-permethrin (40%) compared to *trans*-permethrin (16%). For JAX-EXP, *cis*- and *trans*-permethrin were both detected in 89% of the indoor air samples with median levels of 2.0 and 3.1 ng/m^3^, respectively. These data suggest that children in JAX-EXP were likely exposed to higher levels of permethrin in both indoor and outdoor air than children in the other studies. As permethrin is considered to have low volatility, it is unclear why the JAX-EXP indoor and outdoor air samples had much higher levels of this insecticide compared to the other studies, and more research is needed. 

Pesticide products containing pyrethroids are commonly manufactured as either a single isomer (e.g., *cis-*deltamethrin) or a mixture of isomers (e.g., *cis*- and *trans*-permethrin) [25,31]. *Cis*- and *trans*-isomers of pyrethroids have the same molecular formula, but a different spatial orientation of atoms, and these isomers can have different physical properties and insecticidal and mammalian toxicities [3,31,32]. In these children’s studies, the *cis*- and *trans*-isomers of allethrin and permethrin were measured in several environmental media, however only the *cis*- and *trans*-isomers of permethrin were frequently detected (>40%). Isomeric ratios of permethrin in commercial products are generally manufactured as a mixture with typical *cis*/*trans*-ratios of 0.3 or 0.7 [5,33]. Across these children’s studies, the median *cis/trans*-ratios of permethrin ranged from 0.4 to 1.0, 0.6 to 1.4, 0.7 to 1.6, and 1.1 to 1.3 in the floor wipe, floor dust, indoor air, and duplicate diet solid food samples, respectively. This information suggests that these two isomers are likely degrading at various rates in environmental media at residences. Research has indicated that isomeric degradation rates are likely influenced by environmental factors such as temperature, sunlight, microbial degradation, and location (indoors/outdoors) [34,35]. 

Previous research has indicated that pesticide metabolites used as urinary biomarkers of exposure are also measureable in environmental media [36,37]. The CTEPP-VAC and PEPCOT studies concurrently measured the levels of pyrethroids and their common environmentally-degraded metabolite(s) in environmental media [16,38]. Starr *et al*. [16] measured for 13 parent pyrethroids and their five environmentally-degraded pyrethroid metabolites (3-PBA, 4F-3-PBA, *cis*-DCCA, *trans*-DCCA, DBCA, and CA (3-(2-2-dimethylvinyl)-2,2-dimethylcyclopropane-1-carboxylic acid)) in vacuum dust samples collected at 80 children’s homes (*and 5 child day care centers*) [16]. CA can be a metabolite of allethrin, imiprothrin, phenothrin, prallethrin, resmethrin, and tetramethrin. Of the 13 pyrethroids measured in CTEPP-VAC, all but *cis*- and *trans*-permethrin were detected in less than 37% of the vacuum dust samples. These two permethrin isomers were both detected in 85% of the vacuum dust samples with median levels of 666 and 711 ng/g, respectively. The metabolites 3-PBA, DCCA, DBCA, 4F-3-PBA, and CA were also detected in 67%, 81%, 39%, 8%, and 67%, respectively, of the dust samples. The median levels of 3-PBA, DCCA, DBCA, 4F-3-PBA, and CA were 38, 154, <5, <5, and 29 ng/g, respectively. Wilson *et al.* [38] also reported median concentrations of 3-PBA of 13.6 ng/g, 0.003 ng/cm^2^, and 0.5 ng/m^3^ in carpet dust, hand wipes, and indoor air samples, respectively, collected at 50 PEPCOT children’s homes in North Carolina [7,38]. These studies have provided evidence of the co-occurrence of pyrethroids and their environmentally-degraded metabolites of pyrethroids in several environmental media. These data are important as these same environmentally-degraded metabolites are also used as urinary biomarkers of exposure and could possibly overinflate children’s exposure estimates for pyrethroids.

### 3.2. Urinary Concentrations of Pyrethroid Metabolites

Table 3 presents the detection frequencies and distributions for five pyrethroid metabolites (*cis*-DCCA, *trans*-DCCA, 4-F-3-PBA, DBCA, and 3-PBA) measured in children’s spot urine samples in available studies (CPES (summer and year), CTEPP-OH, JAX-BIO, JAX-EXP, and NHANES). 3-PBA was the most frequently detected (≥67%) pyrethroid metabolites in children’s urine samples among these studies suggesting that the majority of the children were exposed to one or more of the pyrethroids. The NHANES [8], a U.S. representative population survey that included 468 children (1999–2000) and 580 children (2001–2002) ages 6 to 11 years, reported that frequencies of detection were below 48% for all five of these pyrethroid metabolites, except for 3-PBA (>71%). Among these studies, the children’s median urinary 3-PBA concentrations in the JAX-BIO (1.9 ng/mL), JAX-EXP (2.2 ng/mL), and CPES (year; 1.2 ng/mL) were at least four times greater than NHANES (0.03 ng/mL) suggesting that these children were being exposed to higher levels of one or more pyrethroids in different geographical regions of the U.S. Across these studies (at the 75th percentile), the children’s urinary *trans*-DCCA concentrations were between two to four times greater than their urinary *cis*-DCCA concentrations. Barr *et al.* [8] also reported than NHANES urinary *trans*-DCCA to *cis*-DCCA ratio varied greatly, but the majority of the ratios were between 3 and 4 [8]. In the body, the urinary *trans*-DCCA metabolite is produced from exposure to *trans*-permethrin, *trans*-cypermethrin, and/or *trans*-cyfluthrin [25]. Likewise, the urinary *cis*-DCCA metabolite is formed in the body from exposure to *cis*-permethrin, *cis*-cypermethrin, and/or *cis*-cyfluthrin [25]. Studies have shown human volunteers orally administered cypermethrin (1:1 *cis/trans*-ratio), renally eliminated more of the *trans*-isomer dose (78%) than the c*is*-isomer dose (49%) over a 24-h period [39,40]. This information suggests that these children may have been exposed to higher levels of the *trans*-isomer compared to the *cis*-isomer of one or more of the pyrethroids in their environments and/or had greater urinary excretion of the *trans*-DCCA metabolite compared to the *cis*-DCCA metabolite over a day.

The above urinary pyrethroid metabolite data indicate that these children were likely exposed to pyrethroids that are primarily metabolized to 3-PBA and DCCA in the body. Barr *et al.* [8] reported that NHANES urinary *cis*- and *trans*-DCCA concentrations were highly correlated with each other (r = 0.887; *p* < 0.001) and with 3-PBA (r = 0.766; *p* = 0.02) suggesting that the NHANES children were likely exposed predominately to permethrin and cypermethrin. In support of these findings, Lu *et al.* [18] reported frequencies of detection above 80% for only the pyrethroid metabolites, 3-PBA and *trans*-DCCA, in 706 urine samples of CPES children. The co-occurrence of these two metabolites is also a likely indicator of exposure to mainly permethrin and/or cypermethrin. These data suggest that children in these observational exposure measurement studies were likely predominately exposed to permethrin and cypermethrin between 1999 and 2004 in the U.S.

The variability of urinary pyrethroid metabolites in children was examined in the CPES and CTEPP-OH [18,41]. Lu *et al.* [18] measured the urinary pyrethroid metabolite concentrations in spot urine samples collected from 23 CPES children, ages 3–11 years, from Seattle, Washington over a year and showed seasonal differences in the children’s median urinary *trans*-DCCA and 3-PBA concentrations. The children’s urinary *trans*-DCCA and 3-PBA concentrations were at least two and three times lower, respectively, in the summer as compared to the fall, winter or spring months. In addition, they reported that seasonality, dietary intake, and reported residential use of pesticides were significant contributors to the temporal variability of the CPES children’s urinary pyrethroid metabolite concentrations. Riederer *et al.* [42] also showed that diet, but not reported residential pesticide use, was a significant predictor of urinary 3-PBA concentrations in 1999–2002 NHANES children, ages 6–10 years old. The authors suggest that this lack of an association between residential pesticide use and the children’s urinary 3-PBA in NHANES compared to CPES may be related to study design differences such as sampling older children, using spot urine samples, and administering the residential pesticide-use questionnaire a few weeks prior to urine sample collection. Egeghy *et al.* [41] recently showed that spot urine measurements collected over a 48-h period from 13 CTEPP-OH children living in homes with recent pesticide applications had substantial sample-to-sample variability and large differences among these children. The reported intraclass correlation coefficient of reliability (ICC) was 0.69 for the children’s urinary 3-PBA concentrations which indicated that several spot urine samples were probably needed over the 48-h period to provide a reliable estimate of pyrethroid exposure among the CTEPP children [41]. This information is important as a number of past children’s exposure measurement studies have collected only one spot urine sample which is likely not sufficient to provide a reliable estimate of their exposures to pyrethroids over a day or longer (e.g., season or year).

**Table 3:** Concentrations of pyrethroid metabolites (ng/mL) in children’s urine samples by study.

Metabolite	Study ^a^	Year	Age (years)	N ^b^	LOD ^c^(ng/mL)	% Detect	Mean ± SD	Minimum	25th^ d^	50th	75th	95th	Maximum
*cis*-DCCA	NHANES	1999–2000	6–11	468	0.1	47	NR ^h^	NR	NR	< ^i^	0.33	1.7	NR
		2001–2002	6–11	580	0.1	26	NR	NR	NR	<	0.11	0.73	NR
	JAX-BIO ^e^	2001	4–6	201	0.2	87	2.2 ± 7.5	NR	NR	0.60	1.5	7.2	94.4
	JAX-EXP ^e^	2001	4–6	9	0.2	100	NR	NR	NR	0.62	3.9	NR	43
	CPES (summer) ^f^	2003	3–11	724	0.2	35	0.33 ± 1	<	<	<	0.33	NR	15
	CPES (year) ^f^	2003–2004	3–11	706	0.2	44	0.5 ± 2.0	<	<	<	0.70	1.1	38.8
*trans*-DCCA	NHANES	1999–2000	6–11	478	0.4	42	NR	NR	NR	<	0.97	4.2	NR
		2001–2002	6–11	576	0.4	29	NR	NR	NR	<	0.47	2.5	NR
	JAX-BIO	2001	4–6	201	0.1	93	3.6 ± 8.4	NR	NR	1.1	2.8	12.7	85.7
	JAX-EXP	2001	4–6	9	0.2	100	NR	NR	NR	1.1	6.9	NR	61
	CPES (summer)	2003	3–11	724	0.4	71	1.24 ± 2.6	<	0.14	0.38	0.99	NR	25
	CPES (year)	2003–2004	3–11	706	0.4	83	1.4 ± 4.1	<	0.3	1.0	1.5	3.7	81.6
4-F-3-PBA ^g^	JAX-BIO	2001	4–6	201	0.1	70	0.2 ± 0.2	NR	NR	0.1	0.2	0.4	3.0
	JAX-EXP	2001	4–6	9	0.1	56	NR	NR	NR	0.09	0.26	NR	1.7
	CPES (summer)	2003	3–11	724	0.2	2	0.02 ± 0.2	<	<	<	<	NR	3.5
	CPES (year)	2003–2004	3–11	706	0.2	19	0.2 ± 0.4	<	<	<	<	1.2	3.5
DBCA ^g^	CPES (summer)	2003	3–11	724	0.1	2	0.004 ± 0.02	<	<	<	<	NR	0.1
	CPES (year)	2003–2004	3–11	706	0.1	6	0.007 ± 0.03	<	<	<	<	0.04	0.2
3-PBA	NHANES	1999–2000	6–11	483	0.1	72	NR	NR	NR	0.32	1.1	8.6	NR
		2001–2002	6–11	580	0.1	75	NR	NR	NR	0.30	0.76	3.4	NR
	CTEPP-OH	2001	2–5	127	0.2	67	0.9 ± 3.2	<	0.2	0.3	0.7	1.9	33.8
	JAX-BIO	2001	4–6	201	0.2	100	5.0 ± 10.2	NR	NR	1.9	4.2	20.6	89.3
	JAX-EXP	2001	4–6	9	0.5	100	NR	NR	NR	2.2	29	NR	99
	CPES (summer)	2003	3–11	724	0.1	82	1.22 ± 2.4	<	0.22	0.45	0.97	NR	25
	CPES (year)	2003–2004	3–11	706	0.1	94	1.5 ± 3.1	<	0.5	1.2	2.6	4.1	51.4

^a^ CPES (Children’s Pesticide Exposure Study in Washington, summer and year), CTEPP-OH (Children’s Total Exposure to Persistent Pesticides and Other Persistent Organic Pollutants Study, *Ohio only*), JAX-BIO (Biological Monitoring for Organophosphate and Pyrethroid Pesticide Exposures in Children Living in Jacksonville, Florida Study); JAX-EXP (Biological and Environmental Monitoring for Organophosphate and Pyrethroid Pesticide Exposures in Children Living in Jacksonville, Florida Study); and NHANES (National Health and Nutrition Examination Survey); ^b^ Sample level; ^c^ Limit of detection; ^d^ Percentiles; ^e^ A total of 203 children participated in the JAX-BIO and a subset (n = 9) of them also participated in the JAX-EXP; ^f^ Same study; summer season results reported in Lu *et al.* [5] and 12 month period (including summer season) reported in Lu *et al.* [18]; ^g^ For NHANES 1999–2000 and 2001–2002, 4-F-3-PBA and DBCA were detected in less than 3% and 2% of the children’s urine samples, respectively (Barr *et al*. [8]); ^h^ NR = value not reported; ^i ^‘<’ = not detectable.

Assuming steady-state absorption, the children’s estimated cumulative intake doses of pyrethroids in these children’s studies ranged from 0.0002 to 0.002 mg/kg/day with the highest intake doses occurring for JAX-BIO and JAX-EXP (both 0.002 mg/kg/day). The cumulative intake doses were calculated by multiplying the highest reported urinary 3-PBA concentrations for a child in each study by an estimated daily urine excretion rate of 22.4 mL/kg body weight [7]. As no established cumulative oral reference dose for exposure to pyrethroids exist in the U.S. EPA Integrated Risk Information System (IRIS) [43], the children’s cumulative intake doses were compared to single oral reference doses for permethrin (0.05 mg/kg/day) and cypermethrin (0.01 mg/kg/day) since these insecticides were the most frequently detected pyrethroids in residential media. The JAX-EXP and JAX-BIO children’s exposure levels were 25 and 5 times lower than the oral RfDs for permethrin and cypermethrin, respectively. More research is needed to establish health-based standards for children’s cumulative exposures to pyrethroids to ensure that their health is adequately protected.

As environmentally-degraded pyrethroid metabolites have been found in residential environments [16,38], it is unclear whether these same metabolites (e.g., 3-PBA) are substantially absorbed into children’s bodies and contribute to their urinary pyrethroid metabolite concentrations (e.g., 3-PBA)—which could overestimate human exposure estimates for pyrethroids. Data are currently lacking on the toxicokinetics of environmentally-degraded pyrethroid metabolites in exposed humans. However, Huckle *et al.* [44] reported that 3-PBA administered orally or by intraperitoneal injection to several different types of mammalian species (*i.e.*, rats, hamsters, rabbits and guinea pigs) undergoes further metabolism to other products, and these metabolites were primarily renally eliminated. More research is needed to determine if humans directly exposed to environmentally-degraded pyrethroid metabolites are unchanged or are further metabolized to other products in the body. 

### 3.3. Contributions of Pyrethroid Exposure by Route

A few of the children’s exposure measurements studies, CHAMACOS-QEA, CTEPP-OH, JAX-EXP, and PEPCOT, have collected sufficient data (e.g., environmental, biomonitoring, and activity pattern) to quantitatively assess the aggregate or cumulative exposures of children to pyrethroids at home [7,45]. Published data only exist for the CTEPP-OH and the JAX-EXP. Morgan *et al.* [7] estimated the aggregate *everyday* exposures of 57 CTEPP-OH children, ages 2–5 years old, to both *cis*- and *trans-*permethrin through the dietary ingestion, nondietary ingestion, inhalation, and dermal routes and compared these estimates to excreted urinary amounts of 3-PBA. The children’s estimated median aggregate absorbed dose to the combined *cis*- and *trans*-isomers was 4.4 ng/kg/day, and the primary exposure route was determined to be dietary ingestion of solid foods, followed by nondietary ingestion of dust. About 7% of the solid food samples had much higher levels of the combined permethrin isomers which suggests these children were probably consuming certain types of foods (e.g., fruits and vegetables) that contained greater levels of permethrin residues [7]. The children’s estimated median absorbed dose to the combined isomers accounted for greater than 85% of the excreted amount of urinary 3-PBA (5.1 ng/kg/day) suggesting that they were likely mainly exposed to permethrin compared to other possible pyrethroids at their homes. Tulve *et al.* [45] recently estimated the cumulative exposures of the JAX-EXP children, ages 4–6 years old, to several pyrethroids through dietary ingestion, nondietary ingestion, inhalation, and dermal routes. These children lived in homes with reported frequent use of pesticide products. The children’s cumulative exposures to pyrethroids were predicted to be primarily through the dermal route (57%), followed by dietary ingestion route (33%). Using linear regression analysis, a positive relationship (r^2^ = 0.90, *p* < 0.05) was observed between JAX-EXP children’s estimated dermal exposure and their urinary 3-PBA concentrations; however, the authors caution that the results are limited due to the small sample size [45]. This research indicated that dietary ingestion followed by nondietary ingestion were the major exposure routes for these children, except in homes with frequent pesticide applications then dermal followed by dietary ingestion were the major exposure routes. The results of these above studies suggest that children’s exposures to pyrethroids were likely related to their individual eating and activity patterns and whether pyrethroids were applied at their homes. 

### 3.4. Limitations

Several limitations should be considered regarding this review of children’s observational exposure assessment studies. Only the PubMed database was accessed and only U.S. studies conducted between 1999 and 2007 were included. Quantitative comparisons of the data were mainly limited to levels of pyrethroids in environmental and biological media among the studies due to the diverse types of study designs (e.g., number of children, locations, race/ethnicities, socioeconomic status, types of samples collected, and sampling methods). Of these studies, only the NHANES was designed to be representative of the U.S. general population study; nonetheless, it does not collect urinary biomonitoring data for children less than 6 years of age or environmental measurements data. 

Common study limitations observed across the majority of the studies were use of convenience sampling approaches, small sample sizes, cross-sectional designs (24-h or 48-h), limited demographics factors (*i.e.*, ages, races/ethnicities, socioeconomic status), limited types and numbers of environmental samples collected, collection of no or only one spot urine sample per child, a lack of standard methods for collection of samples (except for the HVS3), and a lack of validated questionnaires/diaries. It is recommended that children’s observational exposure measurement studies conducted in the future adopt a common ontology, particularly developing and using standardized sampling and analytical methods and validated questionnaires and diaries so better and more extensive quantitative comparisons of data can be made.

## 4. Conclusions

Residential use of pyrethroids likely varies across the U.S. and over time [46]. However, retail sales data and the amount of pyrethroids applied in residential environments are currently not available [46]. This review indicates that these children were likely exposed to low levels of several pyrethroids, but primarily to permethrin and cypermethrin, from several sources such as diet, dust, and/or surfaces at their residences between 1999 and 2007 in the U.S. Dietary ingestion followed by nondietary ingestion were the major exposure routes for these children, except in homes with frequent pesticide applications, then dermal followed by dietary ingestion were the major routes. Urinary 3-PBA concentration data confirm that the majority of the children sampled were exposed to one or more of the pyrethroids. 

Despite the wealth of data and information generated in these studies, data gaps remain. Children’s exposure assessments could be greatly enhanced by designing future studies to collect additional kinds of data. These data include:

- Levels of pyrethroids and their environmentally-occurring metabolites in individual food items for several consecutive days or a week including seasonally as certain types of foods (*i.e.*, fruits and vegetables) likely contribute to the intermittent dietary exposures of children to current-use pyrethroids,
- Spatiotemporal variability of pyrethroids and their environmentally-degraded metabolites in media (*i.e.*, dust and on surfaces) at residences, since pyrethroid levels can vary substantially within and between rooms, particularly before and after pesticide use,
- Temporal variability of urinary pesticide metabolites over a day and for several consecutive days as there can be substantial intra-individual and inter-individual variability of pyrethroid metabolites in children’s urine samples even over a short period of time (e.g., 48-h period). The volume of the urine void and time of the current and previous urine void should be recorded as this information can be used to calculate the metabolite excretion rate (ng/h) for a pyrethroid which is independent of urine volume,
- Toxicokinetics of environmentally-degraded metabolites in humans (e.g., *in vitro* assays) to determine whether these metabolites are unchanged or are further metabolized to other products in the body before urinary elimination.
- Accurate and more descriptive pesticide product use information including active ingredients as these products can contain single or multiple pyrethroids (including one or more isomers per pesticide), and
- Accurate and more descriptive daily activity patterns of children as their exposures to pesticides are highly dependent on their day-to-day activities such as their individual eating patterns, activity levels, locations they spend their time, hand-to-mouth activity, and object-to-mouth activity.

## Disclaimer

*“ The United States Environmental Protection Agency through its Office of Research and Development has provided administrative review of this article and approved for publication. ”*

## Supplementary Files

Supplementary File 1: PDF-Document (PDF, 70 KB)

## References

[1] U.S. Environmental Protection Agency (EPA) Agrichemicals Food Quality Protection Act—Children and Consumers. http://www.epa.gov/agriculture/factsheets/epa-305-f-00-005ag.html.

[2] Weston D.P., You J., Lydy M.J. (2004). Distribution and toxicity of sediment-associated pesticides in agricultural-dominated water bodies of California’s Central Valley. Environ. Sci. Technol..

[3] Gan J., Spurlock F., Hendley P., Weston D. (2008). Chapter 1: Synthetic Pyrethroid Use Patterns, Properties, and Environmental Effects. Synthetic Pyrethroids, Occurrence and Behavior in Aquatic Environments.

[4] Kaneko H. (2011). Pyrethroids: Mammalian metabolism and toxicity. J. Agric. Food Chem..

[5] Lu C., Barr D.B., Pearson M., Bartell S., Bravo R. (2006). A longitudinal approach to assessing urban and suburban children’s exposure to pyrethroid pesticides. Environ. Health Perspect..

[6] Bradman A., Whitaker D., Quiros L., Castorina R., Henn B.C., Nishiko M., Morgan J.,  Barr D.B., Harnley M., Brisbin J.A. (2007). Pesticides and their metabolites in the homes and urine of farmworker children living in the Salinas Valley, CA. J. Expo. Sci. Environ. Epidemiol..

[7] Morgan M.K., Sheldon L.S., Croghan C.W., Jones P.A., Chuang J.C., Wilson N.K. (2007). An observational study of 127 preschool children at their homes and daycare centers in Ohio: Environmental pathways to *cis*- and *trans*-permethrin exposure. Environ. Res..

[8] Barr D.B., Olsson A.O., Wong L.Y., Udunka S., Baker S.E., Whitehead R.D.,  Magsumboi M.S., Williams B.L., Needham L.L. (2010). Urinary concentrations of metabolites of pyrethroid insecticides in the general U.S. population: National health and nutrition examination survey 1999–2002. Environ. Health Perspect..

[9] Lu C., Schenck F.J., Pearson M.A., Wong J.W. (2010). Assessing children’s dietary pesticide exposure: Direct measurement of pesticide residues in 24-hr duplicate food samples. Environ. Health Perspect..

[10] Leng G., Kuhn K.H., Idel H. (1997). Biological monitoring of pyrethroids in blood and pyrethroid metabolites in urine: Applications and limitations. Sci. Total Environ..

[11] Leng G., Ranft. U., Sugiri D., Hadnagy W., Berger-Preib E., Idel H. (2003). Pyrethroids used indoors—Biological monitoring of exposure to pyrethroids following an indoor pest control operation. Int. J. Hyg. Environ. Health.

[12] Morgan M.K., Sheldon L.S., Croghan C.W., Chuang J.C., Lordo R., Wilson N.K., Lyu C., Brinkman M., Morse N., Chou Y. (2004). A Pilot Study of Children’a Total Exposure to Persistent Pesticides and Other Persistent Organic Pollutants (CTEPP); EPA/600/R-041/193.

[13] Quandt S.A., Arcury T.A., Rao P., Snively B.M., Camann D.E., Doran A.M., Yau A.Y., Hoppin J.A., Jackson D.S. (2004). Agricultural and residential pesticides in wipe samples from farm worker family residences in North Carolina and Virginia. Environ. Health Perspect..

[14] Bouvier G., Vigouroux-Villard A., Blanchard O., Momas I. (2005). Insecticide urinary metabolites in non-occupationally exposed populations. J. Toxicol. Environ. Health B.

[15] Julien R., Adamkiewicz G., Levy J., Bennett D.B., Nishioka M., Spengler J.D. (2008). Pesticide loadings of select organophosphate and pyrethroid pesticides in urban public housing. J. Expo. Sci. Environ. Epidemiol..

[16] Starr J., Graham S., Stout D., Andrews K., Nishioka M. (2008). Pyrethroid pesticides and their metabolites in vacuum cleaner dust collected from homes and day-care centers. Environ. Res..

[17] 17. Tulve N.S., Fortmann R.C., Whitaker D.A., Nishioka M.G., Naeher L.P., Hilliard A. (2008). Multimedia measurements and activity patterns in an observational pilot study of nine young children. J. Expo. Sci. Environ. Epidemiol..

[18] Lu C., Barr D.B., Pearson M., Walker L.A., Bravo R. (2009). The attribution of urban and suburban children’s exposure to synthetic pyrethroid insecticides: A longitudinal assessment. J. Expo. Sci. Environ. Epidemiol..

[19] Naeher L.P., Tulve N.S., Egeghy P.P., Barr D.B., Adetona O., Fortmann R.C., Needham L.L., Bozeman E., Hilliard A., Sheldon L.S. (2010). Organophosphorus and pyrethroid insecticide urinary metabolite concentrations in young children living in a southeastern United States city. Sci. Total Environ..

[20] Chuang J.C., Wilson N.K. (2011). Multiresidue analysis of organophosphate and pyrethroid pesticides in duplicate-diet solid food by pressurized liquid extraction. J. Environ. Sci. Health B.

[21] Quiros-Alcala L., Bradman A., Nishioka M., Harnly M., Hubbard A., McKone T., Ferber J., Eskenazi B. (2011). Pesticides in house dust from urban and farmworker households in California: An observational measurement study. Environ. Health.

[22] O’Rourke M.K., Lizardi P.S., Rogan S.P., Freeman N.C., Aguirre A., Saint C.G. (2000). Pesticide exposure and creatinine variation among young children. J. Expo. Anal. Environ. Epidemiol..

[23] Barr D.B., Wilder L.C., Caudill S.P., Gonzalez A.J., Needham L.L., Pirkle J.L. (2005). Urinary creatinine concentrations in the U.S. population: Implications for urinary biologic monitoring measurements. Environ. Health Perspect..

[24] Fortin M.C., Bouchard M., Carrier G., Dumas P. (2008). Biological monitoring of exposure to pyrethrins and pyrethroids in a metropolitan population of the Province of Quebec, Canada. Environ. Res..

[25] Centers for Disease Control and Prevention (CDC) Fourth National Report on Human Exposure to Environmental Chemicals. http://www.cdc.gov/exposurereport/.

[26] (1994). Standard Practice for Collection of Floor Dust for Chemical Analysis D5438-94. Annual Book of ASTM Standards.

[27] Mercier F., Glorennec P., Thomas O., Le Bot B. (2011). Organic contamination of settled house dust, a review for exposure assessment purposes. Environ. Sci. Technol..

[28] Egeghy P.P., Sheldon L.S., Fortmann R.C., Stout D.M., Tulve N.S., Cohen Hubal E., Melnyk L.J., Morgan M.K., Jones P.A., Whitaker D. (2007). Important Exposure Factors for Children: An Analysis of Laboratory and Observational Field Data Characterizing Cumulative Exposure to Pesticides; EPA 600/R-07/013.

[29] Riederer A.M., Hunter R.E., Hayden S.W., Ryan P.B. (2010). Pyrethroid and organophosphorus pesticides in composite diet samples from Atlanta, USA adults. Environ. Sci. Technol..

[30] Food and Drug Administration (FDA) Total Diet Study Analytical Results. http://www.fda.gov/Food/FoodSafety/FoodContaminantsAdulteration/TotalDietStudy/ucm184293.htm.

[31] Soderland D.M., Clark J.M., Sheets L.P., Mullin L.S., Piccirillo V.J., Sargent D., Stevens J.T., Weiner M.L. (2002). Mechanisms of pyrethroid neurotoxicity: Implications for cumulative risk assessment. Toxicology.

[32] Shafer T.J., Meyer D.A., Crofton K.M. (2005). Developmental neurotoxicity of pyrethroid insecticides: Critical review and future research needs. Environ. Health Perspect..

[33] Couture C., Fortin M.C., Carrier G., Dumas P., Tremblay C., Bouchard M. (2009). Assessment of exposure to pyrethroids and pyrethrins in a rural population of the Monteregie Area, Quebec, Canada. J. Occup. Environ. Hyg..

[34] Liu W., Gan J.J. (2004). Determination of enantiomers of synthetic pyrethroids in water by solid phase microextraction—Enantioselective gas chromatography. J. Agric. Food Chem..

[35] Allan I.J., House W.A., Carter J.E. (2005). Diffusion of the synthetic pyrethroid permethrin into bed-sediments. Environ. Sci. Technol..

[36] Lu C., Bravo R., Caltabiano L.M., Irish R.M., Weerasekera G., Barr D.B. (2005). The presence of dialkylphosphates in fresh fruitjuices: Implication for organophosphorus pesticide exposure and risk assessments. J. Toxicol. Environ. Health A.

[37] Morgan M.K., Sheldon L.S., Croghan C.W., Jones P.A., Robertson G., Chuang J.C.,  Wilson N.K., Lyu C. (2005). Exposures of preschool children to chlorpyrifos and its degradation product 3,5,6-trichloro-2-pyridinol in their everyday environments. J. Expo. Sci. Environ. Epidemiol..

[38] Wilson N.K., Chuang J.C., Hand K.M., Brinkman M., Tefft M.E., Finegold J.K.,  Aysucue L.C., Humphreys N.L., Chapman M.M., Wilson L.K. Organophosphate and Pyrethroid Pesticides in Environmental and Children’s Personal Samples Collected at 50 Homes of Infants and Toddlers. Proceedings of the 2004 Annual Meeting of the International Society of Exposure Analysis.

[39] Eadsforth C.V., Baldwin M.K. (1983). Human dose-excretion studies with the pyrethroid insecticide, cypermethrin. Xenobiotica.

[40] Eadsforth C.V., Bragt P.C., van Sittert N.J. (1988). Human dose-excretion studies with pyrethroid insecticide cypermethrin and alphacypermethrin: Relevance for biological monitoring. Xenobiotica.

[41] Egeghy P.P., Cohen Hubal E.A., Tulve N.S., Melnyk L.J., Morgan M.K., Fortmann R.C., Sheldon L.S. (2011). Review of pesticide urinary biomarker measurements from selected children’s obsevational exposure studies. Int. J. Environ. Public Health.

[42] Riederer A.M., Bartell S.M., Barr D.B., Ryan P.B. (2008). Diet and nondiet predictors of urinary 3-phenoxybenzoic acid in NHANES 1999–2002. Environ. Health Perspect..

[43] Integrated Risk Information System. http://www.epa.gov/IRIS.

[44] Huckle K.R., Hutson D.H., Millburn P. (1981). Species differences in the metabolism of 3-phenoxybenzoic acid. Drug Metab. Dispos..

[45] Tulve N.S., Egeghy P.P., Fortmann R.C., Xue J., Evans J., Whitaker D.A., Croghan C.W. (2011). Methodologies for estimating cumulative human exposures to current-use pyrethroid pesticides. J. Expo. Sci. Environ. Epidemiol..

[46] Kuivila K.M., Hladik M.L., Ingersoll C.G., Kemble N.E., Moran P.W., Calhoun D.L., Nowell L.H., Gilliiom R.J. (2012). Occurrence and potential sources of pyrethroid insecticides in stream sediments from seven U.S. metropolitan areas. Environ. Sci. Technol..

