# Supplementary material for: Children’s Exposures to Pyrethroid Insecticides at Home: A Review of Data Collected in Published Exposure Measurement Studies Conducted in the United States
Source: Int J Environ Res Public Health. 2012 Aug 17;9(8):2964–85. doi: 10.3390/ijerph9082964 (PMC3447599; doi:10.3390/ijerph9082964)
Supplement: Supplementary File 1: — PDF-Document (PDF, 70 KB) [file ijerph-09-02964-s001.pdf]

OPEN ACCESS

International Journal of
Environmental Research and Public Health
ISSN 1660-4601
www.mdpi.com/journal/ijerph

Supplementary Information

Children's Exposures to Pyrethroid Insecticides at Home: A Review of Data Collected in Published Exposure Measurement Studies Conducted in the United States

Marsha K. Morgan

National Exposure Research Laboratory, U.S. EPA, Research Triangle Park, NC 27711, USA;
E-Mail: morgan.marsha@epa.gov; Tel.: +1-919-541-2598; Fax: +1-919-541-0905

Table S1. Levels of pyrethroids measured in floor dust samples (ng/g) collected at children's homes by year of study.

	STUDY [a,b]																	
	**CTEPP-NC [c]** Year = 2000–2001 N = 121 [d]			**CTEPP-OH [c]** Year = 2001 N = 119			**CTEPP-VAC** Year = 2000–2001 N = 85			**CHAMACOS-QEA** Year = 2002 N = 20			**HPHI** Year = 2002–2003 N = 35			**PDLHS [e]** Year = 2006 N = 25		
**Pyrethroid**	% [f]	50th	Range	%	50th	Range	%	50th	Range	%	50th	Range	%	50th	Range	%	50th	Range
**Allethrin [g]**	--- [h]	---	---	---	---	---	8	<	ND-5,862	---	---	---	---	---	---	80	51	ND–289
***cis*-Allethrin**	---	---	---	---	---	---	---	---	---	25	<	ND–2,500	---	---	---	---	---	---
***trans*-Allethrin**	---	---	---	---	---	---	---	---	---	25	<	ND–2,800	---	---	---	---	---	---
**Bifenthrin**	---	---	---	---	---	---	---	---	---	5	<	ND–30	3	<	ND–10	44	<	ND–2,120
**Cyfluthrin**	48	< [i]	ND–4,100	74	195	ND–3,040	20	<	ND–24,607	10	<	ND–300	43	<	ND–48,100	---	---	---
**λ-Cyhalothrin**	---	---	---	---	---	---	7	<	ND–219	20	<	ND–140	3	<	ND–100	---	---	---
**Cypermethrin**	---	---	---	---	---	---	29	<	ND–6,492	40	100	ND–1,500	60	300	ND–5,200	64	587	ND–13,100
**Deltamethrin**	---	---	---	---	---	---	4	<	ND–2,503	5	<	ND–560	9	<	ND–7,000	12	<	ND–16,300
**Esfenvalerate**	---	---	---	---	---	---	7	<	ND–943	5	<	ND–50	29	<	ND–1,200	0	NA	NA
**Fenpropathrin**	---	---	---	---	---	---	0	NA [j]	NA	---	---	---	---	---	---	---	---	---
**Imiprothrin**	---	---	---	---	---	---	4	<	ND–381	---	---	---	---	---	---	4	<	ND–160
**Permethrin [g]**	---	---	---	---	---	---	---	---	---	---	---	---	100	920	100–13,100	---	---	---
***cis*-Permethrin**	100	804	ND–311,000	100	470	17–79,600	85	666	26–30,553	100	150	13–2,900	---	---	---	100	291	12–26,700
***trans*-Permethrin**	100	629	ND–322,000	100	344	17–78,800	85	711	ND–30,420	100	230	22–5,800	---	---	---	100	504	18–46,800
**Phenothrin**	---	---	---	---	---	---	36	<	ND–42,211	20	<	ND–5,500	3	<	ND–100	8	<	ND–116
**Prallethrin**	---	---	---	---	---	---	0	NA	NA	---	---	---	---	---	---	4	<	ND–34
**Resmethrin**	---	---	---	---	---	---	0	NA	NA	0	NA	NA	0	NA	NA	---	---	----
**Tetramethrin**	---	---	---	---	---	---	10	<	ND–4,511	0	NA	NA	6	<	ND–6,000	---	---	---

[a] CHAMACOS-QEA (Center for the Health Assessment of Mothers and Children of Salinas Quantitative Exposure Assessment Study); CTEPP (Children's Total Exposure to Persistent Pesticides and Other Persistent Organic Pollutants Study, *Main Study*), CTEPP-VAC (Vacuum Cleaner Bags from CTEPP *Main Study*); HPHI (Healthy Public Housing Initiative Study); and PDLHS (Pesticides in Dust in Low-Income Households from Agricultural and Urban Communities Study)

[b] Limits of detection: CTEPP-NC and CTEPP-OH (cyfluthrin=20 ng/g; permethrin = 2 ng/g); CTEPP-VAC (range = 1–27 ng/g), except for deltamethrin and resmethrin, (60 ng/g each); CHAMACOS-QEA (range = 1–10 ng/g), except for cyfluthrin, cypermethrin, and deltamethrin (200 ng/g each); HPHI (range = 2–22 ng/g), except for esfenvalerate (50 ng/g) and deltamethrin (500 ng/g); PDLHS (range = 2–20 ng/g, except for deltamethrin (250 ng/g), esfenvalerate (50 ng/g), and imiprothrin (50 ng/g)

[c] Data presented by state (CTEPP-NC and CTEPP-OH)

[d] Sample-level

[e] Data reported for dust collected at urban homes, only

[f] Percentage of samples above the detection limit

[g] Reported as the sum of the *cis*- and *trans*-isomers

[h] '---' = pyrethroid not measured

[i] '<' or ND = not detectable

[j] NA = not applicable

Table S2. Levels of pyrethroids measured in floor wipe samples (ng/cm^2) collected at children's homes by year of study.

	**STUDY [a,b]**																	
	**CTEPP-NC [c] Years = 2000–2001 N=28 [d]**			**CTEPP-OH [c] Year = 2001 N=21**			**JAX-EXP [e] Year = 2001 N=9**			**CFW [f] Year= 2001 N=41**			**CHAMACOS-QEA Year = 2002 N=20**			**HPHI [g] Years = 2002–2003 N=30**		
**Pyrethroid**	%[h]	50th	Range	%	50th	Range	%	50th	Range	%	50th	Range	%	50th	Range	%	50th	Range
***cis*-Allethrin**	--- [i]	---	---	---	---	---	33	<	NR [m]–0.11	---	---	---	20	<	ND–2	---	---	---
***trans*-Allethrin**	---	---	---	---	---	---	33	<	NR–0.05	---	---	---	20	<	ND–2.2	---	---	---
**Bifenthrin**	---	---	---	---	---	---	22	<	NR–0.05	---	---	---	5	<	ND–0.04	3	<	ND–0.01
**Cyfluthrin**	7	< [j]	ND–0.13	10	<	ND–0.08	11	<	NR–3.4	---	---	---	5	<	ND–0.4	43	0.37	0.01–5.7
**λ-Cyhalothrin**	---	---	---	---	---	---	11	<	NR–0.12	---	---	---	5	<	ND–0.03	23	0.17	0.02–0.8
**Cypermethrin**	---	---	---	---	---	---	67	0.7	NR–18	---	---	---	40	<	ND–2.8	63	0.38	0.06–6.3
**Deltamethrin**	---	---	---	---	---	---	0	NA [l]	NA	---	---	---	0	NA	NA	7	0.34	0.2–0.5
**Esfenvalerate**	---	---	---	---	---	---	0	NA	NA	12	NR	0.48-9.4	0	NA	NA	50	0.1	0.02–2.7
**Permethrin [k]**	---	---	---	---	---	---	---	---	---	---	---	---	---	---	---	93	0.60	0.07–7.5
***cis*-Permethrin**	93	0.04	ND–0.87	71	0.01	ND–5.2	67	0.04	NR–9.8	66	NR	0.14-32	85	0.10	ND–1.7	---	---	---
***trans*-Permethrin**	93	0.04	ND–1.0	71	0.01	ND–5.2	78	0.05	NR–14	93	NR	0.08-49	95	0.23	ND–3.6	---	---	---
**Phenothrin**	---	---	---	---	---	---	33	<	NR–1.4	---	---	---	15	<	ND–3.5	3	<	ND–0.04
**Resmethrin**	---	---	---	---	---	---	---	---	---	---	---	---	0	NA	NA	3	<	ND–0.01
**Tetramethrin**	---	---	---	---	---	---	33	<	NR–0.28	---	---	---	0	NA	NA	10	0.82	0.01–0.9

[a] CHAMACOS-QEA (Center for the Health Assessment of Mothers and Children of Salinas Quantitative Exposure Assessment Study); CFW (Children of Farm workers Study); CTEPP (Children's Total Exposure to Persistent Pesticides and Other Persistent Organic Pollutants Study), HPHI (Healthy Public Housing Initiative Study); and JAX-EXP (Biological and Environmental Monitoring for Organophosphate and Pyrethroid Pesticide Exposures in Children Living in Jacksonville, Florida Study)

[b] Limits of detection: CTEPP-NC and CTEPP-OH (cyfluthrin=0.007 ng/cm^2; permethrin= 0.001 ng/cm^2); JAX-EXP (range = 0.005-0.016 ng/cm^2); CFW (range 0.005 to 0.03 ng/cm^2); CHAMACOS-QEA (range = 0.0001-0.001 ng/cm^2, except for cyfluthrin and cypermethrin (0.025 ng/cm^2) and deltamethrin (0.020 ng/cm^2); HPHI (range = 0.001-0.027 ng/cm^2), except for deltamethrin (0.27 ng/ cm^2)

[c] Data presented by state (CTEPP-NC and CTEPP-OH)

[d] Sample-level

[e] Data reported for wipes collected in child's main play area inside home

[f] Median values not reported; mean±standard deviation values for *cis*- and *trans*-permethrin were 3.1±6.6 ng/cm^2 and 3.4±8.6 ng/cm^2, respectively

[g] Data reported for floor wipes collected in living room

[h] Percentage of samples above the detection limit

[i] '---' = pyrethroid not measured

[j] '<' or ND = not detectable

[k] Reported as the sum of the *cis*- and *trans*-isomers

[l] NA = not applicable

[m] NR = minimum value was not reported

Table S3. Levels of pyrethroids measured in children's duplicate diet solid food samples (ng/g) by year of study.

	STUDY [a,b,c]																	
	CTEPP-NC [d] Years = 2000–2001 N=128[e]			CTEPP-OH [d] Year = 2001 N=125			JAX-EXP Year = 2001 N=9			CHAMACOS-QEA Year = 2002 N=20			PEPCOT-O [e] Years = 2003–2005 N=142			PEPCOT-Y [f] Years =2003–2005 N=136		
**Pyrethroid**	% [g]	50th	Range	%	50th	Range	%	50th	Range	%	50th	Range	%	50th	Range	%	50th	Range
***cis*-Allethrin**	--- [h]	---	---	---	---	---	0	NA [j]	NA	---	---	---	---	---	---	---	---	---
***trans*-Allethrin**	---	---	---	---	---	---	0	NA	NA	---	---	---	---	---	---	---	---	---
**Bifenthrin**	---	---	---	---	---	---	33	<	NR [k]–1.3	---	---	---	<20 [l]	NR	NR	<20	NR	NR
**Cyfluthrin**	6	< [i]	ND–4.7	3	<	ND–22	22	<	NR–3.6	---	---	---	<20	NR	NR	<20	NR	NR
**λ-Cyhalothrin**	---	---	---	---	---	---	0	NA	NA	---	---	---	<20	NR	NR	<20	NR	NR
**Cypermethrin**	---	---	---	---	---	---	67	2.3	NR–9.5	---	---	---	<20	NR	NR	<20	NR	NR
**Deltamethrin**	---	---	---	---	---	---	11	<	NR–13	---	---	---	<20	NR	NR	<20	NR	NR
**Esfenvalerate**	---	---	---	---	---	---	0	NA	NA	---	---	---	<20	NR	NR	<20	NR	NR
**Fenpropathrin**	---	---	---	---	---	---	---	---	---	---	---	---	<20	NR	NR	<20	NR	NR
**Fenvalerate**	---	---	---	---	---	---	---	---	---	---	---	---	<20	NR	NR	<20	NR	NR
***cis*-Permethrin**	46	<	ND–81	31	<	ND–560	78	0.29	NR–13	0	NA	NA	>50	0.15	0.04-4.4	>50	0.10	0.04–4.1
***trans*-Permethrin**	46	<	ND–70	31	<	ND–448	78	0.22	NR–22	0	NA	NA	>50	0.14	0.04-6.2	>50	0.09	0.04–4.1
**Phenothrin**	---	---	---	---	---	---	11	<	NR–0.11	---	---	---	<20	NR	NR	<20	NR	NR
**Resmethrin**	---	---	---	---	---	---	---	---	---	---	---	---	<20	NR	NR	<20	NR	NR
**Tetramethrin**	---	---	---	---	---	---	22	<	NR–0.13	---	---	---	<20	NR	NR	<20	NR	NR

[a] CHAMACOS-QEA (Center for the Health Assessment of Mothers and Children of Salinas Quantitative Exposure Assessment Study); CTEPP (Children's Total Exposure to Persistent Pesticides and Other Persistent Organic Pollutants Study), JAX-EXP (Biological and Environmental Monitoring for Organophosphate and Pyrethroid Pesticide Exposures in Children Living in Jacksonville, Florida Study), and PEPCOT (The Pesticide Exposures of Preschool Children Over Time Study).

[b] Limits of detection: CTEPP-NC and CTEPP-OH (cyfluthrin = 0.83 ng/g; permethrin = 0.08 ng/g); CHAMACOS-QEA (permethrin = 4.5 ng/g); JAX-EXP (range = 0.02–0.4 ng/g); PEPCOT (range = 0.1–0.5 ng/g)

[c] Only CTEPP-NC, CTEPP-OH, and CHAMCOS-QEA studies quantified the levels of pyrethroids (permethrin and/or cyfluthrin) in liquid food samples; these pyrethroids were detected in less than 20% of these samples

[d] Data presented by state (CTEPP-NC and CTEPP-OH)

[e] Sample-level

[f] Data presented for older sibling (PEPCOT-O) and younger sibling (PEPCOT-Y) from the same household. Data summarized over the three year collection period (2003–2005)

[g] Percentage of samples above the detection limit

[h] '---' = pyrethroid not measured

[i] '<' or ND = not detectable

[j] NA = not applicable

[k] NR = minimum value was not reported.

[l] Frequencies of detection were reported to be generally <20% for all measured pyrethroids (individual data not reported), except for permethrin (Chuang and Wilson [20])

Table S4. Levels of pyrethroids measured in indoor air samples (ng/m^3) collected from children's homes by year of study.

	STUDY [a,b,c]											
	CTEPP-NC [d] Years = 2000–2001 N=128 [e]			CTEPP-OH [d] Year = 2001 N=125			JAX-EXP Year = 2001 N=9			CHAMACOS-QEA Year = 2002 N=20		
**Pyrethroid**	% [f]	50th	Range	%	50th	Range	%	50th	Range	%	50th	Range
***cis*-Allethrin**	--- [g]	---	---	---	---	---	33	<	NR [j]–74	15	<	ND–63
***trans*-Allethrin**	---	---	---	---	---	---	33	<	NR–38	15	<	ND–61
**Bifenthrin**	---	---	---	---	---	---	11	<	NR–3.0	5	<	ND–3.1
**Cyfluthrin**	4	< [h]	ND–183	2	<	ND–9.4	11	<	NR–5.5	0	NA	NA
**λ-Cyhalothrin**	---	---	---	---	---	---	0	NA [i]	NA	0	NA	NA
**Cypermethrin**	---	---	---	---	---	---	22	<	NR–100	5	<	ND–380
**Deltamethrin**	---	---	---	---	---	---	0	NA	NA	0	NA	NA
**Esfenvalerate**	---	---	---	---	---	---	0	NA	NA	0	NA	NA
***cis*-Permethrin**	66	0.58	ND–34	22	<	ND–5.4	89	2.0	NR–92	40	<	ND–8.2
***trans*-Permethrin**	66	0.36	ND–41	18	<	ND–6.8	89	3.1	NR–130	16	<	ND–11
**Phenothrin**	---	---	---	---	---	---	11	<	NR–4.2	10	<	ND–96
**Resmethrin**	---	---	---	---	---	---	---	---	---	0	NA	NA
**Tetramethrin**	---	---	---	---	---	---	22	0.15	NR–63	0	NA	NA

[a] CHAMACOS-QEA (Center for the Health Assessment of Mothers and Children of Salinas Quantitative Exposure Assessment Study); CTEPP (Children's Total Exposure to Persistent Pesticides and Other Persistent Organic Pollutants Study), and JAX-EXP (Biological and Environmental Monitoring for Organophosphate and Pyrethroid Pesticide Exposures in Children Living in Jacksonville, Florida Study)

[b] Limits of detection: CTEPP-NC and CTEPP-OH (cyfluthrin=0.9 ng/m^3; permethrin= 0.09 ng/m^3 (NC) and 0.3 ng/cm^3 (OH)); JAX-EXP(range 0.4-3.0 ng/m^3); CHAMACOS-QEA (range 0.28-1.11 ng/m^3), except for cyfluthrin and cypermethrin (27.8 ng/m^3), deltamethrin(13.9 ng/m^3), and esfenvalerate (6.9 ng/m^3)

[c] All studies collected outdoor air samples. The percentage of detected pyrethroids were < 31% in the outdoor air samples except for JAX-EXPFor JAX-EXP, c*is*- and *trans*-permethrin were both detected in 100% of the nine air samples; median concentrations were 2.1 and 2.5 ng/m^3, respectively

[d] Data presented by state (CTEPP-NC and CTEPP-OH)

[e] Sample-level

[f] Percentage of samples above the detection limit

[g] '---' = pyrethroid not measured

[h] '<' or ND = not detectable

[i] NA = not applicable

[j] NR = minimum value was not reported.

© 2012 by the authors; licensee MDPI, Basel, Switzerland. This article is an open access article distributed under the terms and conditions of the Creative Commons Attribution license (http://creativecommons.org/licenses/by/3.0/).
